# Iliaco-enteric fistula after robot-assisted comprehensive surgical staging of endometrial cancer: a case report

**DOI:** 10.1007/s11701-018-0864-8

**Published:** 2018-08-20

**Authors:** Sahar Salehi, Alireza Daryapeyma, Chikako Suzuki, Ulrika Joneborg, Henrik Falconer

**Affiliations:** 1 Department of Women’s and Children’s Health, Karolinska Institutet and Theme Cancer, Karolinska University Hospital, 17176 Stockholm, Sweden; 2 Department of Vascular Surgery, Karolinska Institute and Karolinska University Hospital, Stockholm, Sweden; 3 Department of Molecular Medicine and Surgery, Department of Diagnostic Radiology, Karolinska Institute and Karolinska University Hospital, Stockholm, Sweden

**Keywords:** Endometrial cancer, Hematochezia, Iliac artery pseudoaneurysm, Iliaco-enteric fistula, Lymphadenectomy

## Abstract

Fistula formation between bowel and blood-vessel is a very rare complication after intraabdominal surgery. We report a case of iliaco-enteric fistula following robot-assisted surgical staging of endometrial cancer. A 71-year-old woman subjected to comprehensive endometrial cancer staging presented with hematochezia 35 days postoperatively. A retroperitoneal right-sided abscess and an iliaco-enteric fistula was confirmed upon imaging. The patient received endovascular repair of the aneurysm in her right common iliac artery and the segments of the small bowel containing the fistula were resected via laparotomy. If a patient presents with new onset postoperative hematochezia after pelvic and/or paraaortic lymphadenectomy, fistulation between bowel and the major abdominal blood vessels should be part of the differential diagnoses.

## Introduction

Endometrial cancer is the most common gynecologic malignancy in the western world [1]. Surgical staging of presumed early stage endometrial cancer includes lymphadenectomy [2]. Minimally invasive surgery is today the preferred surgical modality with robot-assisted surgery gaining ground over traditional laparoscopy [3].

We present an unusual postoperative complication, never encountered previously at our department. We have neither found any previous reports in the literature of a similar case after minimally invasive gynecologic oncology surgery.

## Case summary

A 71-year-old woman with presumed International Federation of Gynecology and Obstetrics (FIGO) stage I serous adenocarcinoma of the endometrium, was scheduled for hysterectomy and bilateral salpingo-oophorectomy with pelvic and infrarenal paraaortic lymphadenectomy by robot-assisted laparoscopy with dual docking [4]. Her medical history included obesity (body mass index 30 kg/m^2^), hypertension and hypothyroidism. The surgery was uneventful except for difficulties to gain access to the paraaortic field because of small bowel preventing exposure. Only a minimal extirpation of interaortocaval nodes below the inferior mesenteric artery was performed in addition to the hysterectomy, bilateral salpingo-oophorectomy and pelvic lymphadenectomy. Further the hysterectomy specimen was extirpated through a small laparotomy due to vaginal stenosis. Operation time was 243 min and blood loss estimated to 50 mL. The postoperative course was normal and the patient was discharged the second day after surgery. Final histopathology showed stage IB serous adenocarcinoma of the endometrium with a total of 59 pelvic and 3 paraaortic lymph nodes. Adjuvant chemotherapy was recommended but the patient declined further treatment.

On the 35th morning after surgery the patient presented at the emergency department with hematochezia. She had further noted several black stools during the night and in the morning, they had turned red. No hematemesis or hematuria was reported. She was circulatory stable without fever or abdominal pain [hemoglobin 101 g/L (normal range 117–153), serum-creatinine 99 µmol/L (normal range < 90), serum C-reactive protein 300 mg/L (normal range < 3), blood-leukocyte particle concentration 35.1 × 10^9^/L (normal range 3.5–8.8), blood-thrombocyte particle concentration 588 × 10^9^/L (normal range 165–387)]. Computed tomography (CT) of the abdomen revealed a 15 cm right-sided retroperitoneal abscess extending from the pelvis to the kidney, and a possible small aneurysm in the right CIA. The abscess communicated with the small bowel but it was not possible to determine on which level. CT-angiography confirmed a 6 mm aneurysm with suspicious extravasation. Broad spectrum antibiotics was initiated.

**Fig. 1:**
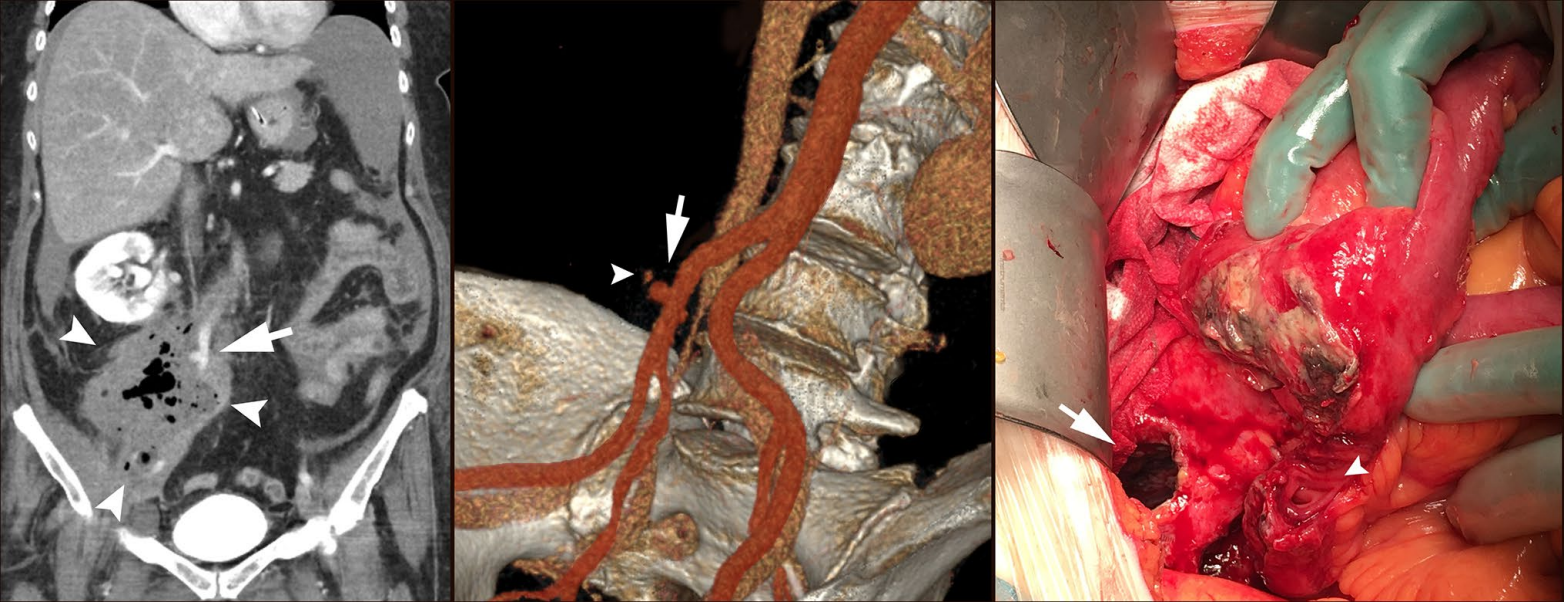
Left: coronal abdominal computed tomography with right sided retroperitoneal abscess (small arrows) and pseudoaneurysm (large arrow). Middle: 3-D volume computed tomography reconstruction of abdominal vessels, pseudoaneurysm in right common iliac artery (large arrow) and minimal extravasation (small arrow). Right: per-operative laparotomy, opening of the abscess (large arrow) after small bowel adherent to the abscess has been retracted with one of the fistula in the ileum is presenting with visible mucosa (small arrow)

The vascular surgeons decided on endovascular repair with percutaneous placement of a self-expanding stent graft (11 mm × 50 mm, Viabahn^®^, W.L. Gore, Flagstaff, AZ, USA), which was performed on postoperative day 36. Explorative laparotomy with the intent to resect involved small bowel segments and protective stoma was then performed. The option of primary anastomosis of the bowel was not considered because of a very low plasma-albumin 13 g/L (normal range 36–48) and hence risk of anastomotic leakage. In the event of duodenal involvement, conservative management with abdominal drainage was planned. A right sided 15 cm retroperitoneal abscess was confirmed and the intraperitoneal surfaces were unaffected by inflammation. The duodenum was unaffected but two segments of the ileum were communicating with the abscess. The mesentery of the right colon covering the hematoma/abscess was inflammatory and stiff. The abscess was incised and a necrotic cavity with foul odor and old blood clots was entered (Fig. 1). The right ureter could not be visualized and neither the CIA, but the latter was easily palpated. The necrotic cavity was thoroughly irrigated. The two fistulas of the small bowel were resected with a total of 1 m small bowel together with the caecum to allow mobilization of the right colon for a mucus fistula in addition to a diverting ileo-stoma. Multiple abdominal drains were placed and the abdomen closed. A percutaneous unilateral nephrostomy was placed. Postoperatively, the patient suffered a deep vein thrombosis in her right arm following placement of a peripheral central vein catheter and was treated with high dose low molecular weight heparin but otherwise she recovered well and was discharged after 19 days.

Within 2 months after laparotomy, the patient was readmitted twice because of high liquid output in the ileostomy resulting in deranged serum electrolytes and impaired renal function. Three months’ post laparotomy, pyelography showed a patent right ureter and the nephrostomy was successfully removed. The ileostomy and mucous fistula of the ascending colon was reversed without the need of laparotomy and the patient was later discharged without complications. The vascular stent remains in situ without any signs of infection or need of treatment with antibiotics. Follow-up with CT-angiography is scheduled.

## Discussion

Isolated iliaco-enteric fistula, is a very rare diagnosis with unclear pathogenesis. The majority of reported cases of iliaco-enteric fistulas have not been associated with prior vascular surgery and predisposing factors include male gender, pelvic surgery, malignancy and infection [5]. Fistula to the colon is most common followed by small bowel and rectum. The common iliac artery (CIA) is the most frequently involved iliac vessel.

The cause of the iliaco-enteric fistula in our patient is most probably occult thermal injury to the small bowel at time of primary surgery with abscess formation where the infection facilitated creation of a small pseudoaneurysm. Though less likely, extensive lymphadenectomy resulting in an iatrogenic pseudoaneurysm in the CIA with secondary hematoma and erosion to the small bowel poses an alternative explanation.

The options for vascular repair included open, definitive repair of the iliac pseudoaneurysm using a venous conduit or endovascular repair with a stent graft. The latter can be considered either a bridging procedure or definite repair with the advantage of maintaining distal perfusion while allowing for thorough debridement to avoid exposing vascular anastomoses to an infected surgical field. Given the extent of the retroperitoneal abscess and the level of contamination, a bridging approach with placement of stent graft in the common iliac was chosen. This approach has been used successfully for the treatment of infected arterial pseudoaneurysms in several situations [6–8]. The need for further vascular intervention in our patient is dictated by the presence of infection in the affected area and the suspicion of graft infection in the right common iliac artery upon follow-up. Previous case reports have shown that the endovascular approach chosen in our patient combined with surgical debridement and long term antibiotic use can be considered a safe long term solution [9, 10].

## Conclusion

Cancer surgery, even though routine, might result in unexpected, serious and life-threatening complications. Urgent multidisciplinary management is then required, why these procedures should preferably be performed at a tertiary referral center with such resources.

If a patient presents with new onset postoperative hematochezia after pelvic and/or paraaortic lymphadenectomy, fistulation to the abdominal vessels should be part of the differential diagnoses.
